# Replication and Functional Prediction of Two GWAS-Reported SNPs Located on RAD50 Gene Associated with Asthma in Pakistani Children

**DOI:** 10.21203/rs.3.rs-7456490/v1

**Published:** 2025-09-17

**Authors:** Muhammad Usman Ghani, Muhammad Farooq Sabar, Iqbal Bano, Qurat-ul ain, Mariam Shahid, Zohair Mehdi, Muhammad Umer Khan

**Affiliations:** University of the Punjab; University of the Punjab; The Children's Hospital, The Institute of Child Health; University of the Punjab; University of the Punjab; University of the Punjab; The University of Lahore

**Keywords:** Asthma, Genetics, Genome-Wide Association Study, Inflammation, Pakistan, SNP

## Abstract

**Background:**

Genome-wide association studies (GWAS) have indicated that several single nucleotide variants (SNVs) of the *RAD50* gene are significantly associated with childhood-onset asthma. However, the biological role of *RAD50*, and its genomic variants that predispose individuals to asthma, remains unclear. This case-control study aimed to investigate the association of two Single nucleotide polymorphisms (SNPs) rs2244012, and rs6871536 of *RAD50* with asthma susceptibility using experimental and computational tools.

**Methods:**

The case-control study involved 355 participants: “176 asthma cases [mean age (sd) = 8.91 ±3.05] and 179 healthy controls [mean age (sd) = 11.10 ±8.86] from local Punjabi population of Pakistan. The SNPs were analyzed using a modified single base extension method. The allelic association with asthma and linkage disequilibrium (LD) between the two main SNPs were performed using the SHEsis tool. SNPStats was used to assess the association of SNPs under genotypic models and interaction with non-genetic factors. The LD calculator of ENSEMBL employed for the identification of proxy SNPs in high LD (r^2 > 0.97) to main SNPs. Additionally, HaploReg(v4.1) was utilized to gauge the impact of SNPs on genomic regulations.

**Results:**

In current study, both SNPs were found to have a significant association (p-value <0.05) with childhood-onset asthma development under allelic and genotypic models. The alternative “G” allele of rs2244012 is shown to modify two regulatory motifs: Nrf-2 and Zbtb12, while the alternative “C” allele of rs6871536 is predicted to alter the OSF-2 motif. Moreover, 10 SNVs proximal to rs2244012 and 21 SNVs near rs6871536 are in high LD in the Punjabi population of Lahore, Pakistan (PJL). These proxy/high-LD SNVs also displayed the potential to change DNA regulatory motifs.

**Conclusion:**

the rs2244012, and rs6871536 variants of *RAD50* gene are significantly association with childhood asthma in Pakistan. Despite being intronic variants, it is our inference that these two SNPs have the potential to either independently or synergistically regulate inflammatory responses via nearby SNVs.

## Background

Asthma is a prevalent inflammatory disorder of the airways, governed by T-lymphocytes. This disorder arises from a complex interplay of risk factors, encompassing both genetic and environmental elements. The condition manifests due to excessive mucus production, inflammation, and airway wall remodeling, culminating in bronchial hyper-reactivity and airway obstruction [1–4]. Approximately a quatar of a billion people worldwide suffer from asthma, and the global burden continues to increase, particularly in low and middle-income countries [5–7].

Several genes within the cytokine gene cluster on chromosome 5, specifically *IL3*, *IL4*, *CSF2*, *TSLP*, *IL5*, *RAD50*, and *IL13*, are recognized as potential asthma susceptibility genes due to their roles in inflammatory regulation among sensitized asthma patients. *RAD50*, located in the 5q31 genomic region of this gene cluster between the *IL5* and *IL13* genes, encodes a protein responsible for DNA double-strand break repair. Even though the expression of this protein is relatively low in most tissues, making its direct function related to asthma unclear, the MRN complex (comprising *MRE11*, *RAD50*, and *NBS1*) plays a role in the somatic hypermutation and gene conversion of the immunoglobulin region [8–10]. Despite limited evidence regarding the gene expression of *RAD50* in airways and its direct association with asthma, genome wide association studies (GWAS) and candidate gene studies have identified genomic variants of this gene as being significantly linked to the disease. Consequently, further exploration is required to understand the functional relationship of this gene with asthma [11, 12]. At the 3' end of the *RAD50* gene lies a locus control region (LCR) that contains numerous conserved non-coding sequences and enhancer elements. These elements regulate the neighboring *IL4* and *IL13* genes. SNPs located within this LCR have been linked to asthma and variations in total serum IgE levels [13]. Li et al. conducted a GWAS on asthma-related traits in 473 asthma patients from the TENOR study and 1,892 general population controls and identified the *RAD50-IL13* region as having the most robust association, with multiple SNPs significantly linked to asthma susceptibility. Notably, the SNP rs2244012 in the *RAD50* gene, in conjunction with the rs1063355 SNP in the *HLA-DQB1* region, exhibited a strong association with asthma. Additionally, imputation pinpointed several other SNPs in the *RAD50-IL13* region on chromosome 5q31 that were related to the disease [8]. Bønnelykke et al. conducted a GWAS on patients with the asthma phenotype, characterized by recurrent, severe exacerbations between 2 and 6 years of age. The study included a total of 1,173 cases and 2,522 controls, identifying five loci (*GSDMB*, *IL33*, *RAD50*, *IL1RL1* and *CDHR3*). Within *RAD50*, rs6871536 was identified as potentially associated with asthma (OR = 1.44, P = 1.7 × 10 – 9) [14].

We carried out a case-control study to assess the role of the asthma susceptibility SNP variants (rs2244012 and rs6871536) of the *RAD50* gene, as predicted by GWAS, in relation to asthma pathogenesis within a scarcely studied Pakistani population. We also employed bioinformatics tools to investigate the potential effects of these intronic variants on the regulation of inflammatory and immunogenic pathways.

## Methods

Our case-control study involved 355 participants: 176 asthmatic children (cases) and 179 healthy children (controls) from Pakistan. Patients with asthma, as diagnosed by physicians, were recruited from the asthma unit of The Children's Hospital and Institute of Child Health (CH & ICH), Lahore. For the purposes of this retrospective investigation, only pediatrically healthy children were enrolled.

We obtained written informed consent from the guardians of both the cases and controls. The study received ethical approval from the institutional review board of CH & ICH, Lahore, and the bioethical committee of The University of the Punjab, Lahore.

Whole blood samples from both groups were subjected to genomic DNA extraction using the phenol-chloroform method [15]. We determined the alleles of the target SNPs (rs2244012 and rs6871536) using a modified single base extension (SBE)/SNaPshot genotyping method, as described in our previously published work [16]. Both SNPs were amplified as two amplicons in a multiplex PCR, and the amplified amplicons were used as templates in a modified SBE/SNaPshot reaction for SNP identification, which was performed on ABI-3130XL genetic analyzer.

Statistical analysis in case-control study: Linkage disequilibrium (LD) between SNPs and their association with the disease in the allelic model (single site analysis) was determined through SHEsis Pluse [17]. It is an online platform for multi-allelic association test. The input data was prepared according the given format and default parameters were used for analysis. The allelic model tests the association between each allele of the target SNP and disease to evaluate if a specific allele is statistically more frequent in one of the groups. In SHEsis Plus, the effect allele will be considered as the allele with the lowest frequency in the study population. Another online tool, SNPStats [18], was used to determine the association of these SNPs in various inheritance (genotypic) models. The selection of the statistical model that best fit the data in this study was based on the Akaike information Criteria (AIC) value. The genotypic model evaluates the effect of the different genotypes of a single genetic variant (i.e., the combination of alleles present at a specific locus for a specific individual) in relation to a phenotype. The data was arranged in an excel file for both softwares according to data guidelines provided on the website. No paramerters were changed and the analysis was adjusted by sex as a covariate. SNPStats also helped to analyze the interaction of SNP variants with various non-genetic factors (Gender, Onset Age, Residential Area, Consanguineous Marriage, Pre-natal Smoke Exposure). This was done under the statistical model that observed most suitable for genotypic association analysis, with a p-value < 0.05 considered indicative of a significant association.

### Bioinformatics Analysis:

Both SNPs of the *RAD50* gene were further investigated using Linkage Disequilibrium Calculator hosted by ENSEMBL(http://useast.ensembl.org/Homo_sapiens/Tools/LD). Our goal was to identify SNVs in strong LD (r^2 > 0.97) with rs2244012 and rs6871536, located within 9kb upstream and downstream of their genomic position (GRCh38.p13). We narrowed our search to SNVs specific to the Punjabi population of Lahore, Pakistan (1000GENOMES:phase_3:PJL). SNVs found to be in high LD with our target SNPs (rs2244012 and rs6871536) were subsequently examined in HaploReg v4.1 to assess their potential impacts on the target SNPs of the *RAD50* gene.

## Results

The case group studied in this research included 67.92% male participant out of which 50% belonged to urban residential areas. Cousin marriage among parents of cases was 63.29% and 52.38% had mother's smoking exposure during gestation. Similarly, controls include 52.1% males, 69.23% urban residential area, 45.13% cousin marriage, and 38.19% mother's smoking exposure during gestation. The demographic characteristics of study participants also given in (Table 1).

The statistical analysis of targeted genomic variants predicted both SNPs (rs2244012, rs6871536) to be significantly associated with asthma disease in both allelic (rs2244012 p-value 0.0014, rs6871536 p-value 0.020) and genotypic models (rs2244012 p-value 0.0039, rs6871536 p-value 0.038) as mentioned in Table 2, Table 3, and Table 4.

Genotypic analysis of rs2244012, adjusting for sex, revealed significant associations under dominant (P = 0.0039), codominant (P = 0.009), and recessive (P = 0.044) inheritance models in our sample population (Table 3). The dominant model was predicted to best fit model based on lowest AIC value. The dominant model suggests that both “G/G” homozygous and “A/G” heterozygous genotypes of rs2244012 may enhance childhood asthma onset susceptibility in studied population. When adjusting for sex, genotypic analysis of rs6871536 revealed significant associations only under the recessive model (p-value = 0.038) (Table 4). The recessive model, with the lowest AIC value of 408.1, was optimal, indicating that only the “C/C” homozygous genotype of rs6871536 heightens asthma risk. The association of target SNPs with covariates is given in Table 5.

The main target SNPs of *RAD-50* gene are in high LD (r^2^ ≥ 0.97) in “Punjabi in Lahore-Pakistan (1000GENOMES:phase_3:**PJL**)” and their alternative alleles are known to alter the DNA regulatory motifs as described in Table 6.

The LD analysis of rs2244012 in ENSEMBL predicted that 10 SNVs are in strong LD (r^2^ > 0.97) to rs2244012 (including rs10079653, rs62383710, rs34776903, rs2706345, rs2243677, rs2706347, rs2706348, rs2706349, rs56668723, rs62383714) with high LD (r^2 > 0.97) in “Punjabi in Lahore-Pakistan (1000GENOMES:phase_3:**PJL**)” population. The HaploReg analysis of these SNVs is presented in Supplementary Table 1. HaploReg pointed out the effect of these SNVs on DNA regulatory motifs.

The LD analysis of rs6871536 in ENSEMBL predicted that 21 SNVs are in strong LD (r^2^ > 0.97) to RAD-50.rs6871536 (like rs6873732, rs6874184, rs62383757, rs62383758, rs3798135, rs3798134, rs56183820, rs6873897, rs12332204, rs6596087, rs6866095, rs10052993, rs12653750, rs2040703, rs2040704, rs11420290, rs377716981, rs6872131, rs2074369, rs7737470, rs11308531) in “Punjabi in Lahore-Pakistan (1000GENOMES:phase_3:**PJL**)” Population. The HaploReg analysis of these SNVs is presented in Supplementary Table 2.

## Discussion:

Asthma is a heterogeneous disorder which affects the lungs and have a different onset age. Usually it is categorized as childhood and adult onset asthma with various cutoff points such as 12, 16, 18, or more recently identified 40y and onward referred as late-onset phenotype [19]. *RAD50* is a plausible candidate gene implicated in asthma pathogenesis. Variants within *RAD50* add to the complexity of factors in the cytokine gene cluster on chromosome 5q31, underscoring the necessity for thorough research [20, 21]. The two SNPs from the *RAD50* gene, rs2244012 and rs6871536, which are featured in this study, are situated in two introns of *RAD50*. They have been previously identified as asthma susceptibility variants in prior GWAS [8, 14]. In this study, the alternative allele “G” of *RAD50*′s rs2244012 showed a significant association with asthma in the allelic model (P = 0.0014). The odds ratio for this allele was higher in asthma patients, with an OR of 1.768 (1.245 ~ 2.510) as determined by the SHEsis analysis (Table 2). The dominant model was predicted to be the best fit in the genotypic analysis, indicating that both the G/G (homozygous) and A/G (heterozygous) genotypes enhance the risk of childhood-onset asthma (Table 3). A single copy of the altered allele “G” increases the risk of the disease, and having two copies of the altered allele (G/G genotype) may increase the risk even more.

Similarly, the risk allele “C” of the rs6871536 SNP variant was more common in asthma patients compared to controls in the studied population. This allele was significantly associated with asthma in the allelic model (p-value = 0.020) with an OR of 1.522 (1.066 ~ 2.174) (Table 2), suggesting that this variants may independently associated with the risk of childhood-onset asthma in Pakistan. The recessive model was found to be the best fit and statistically significant in genotyping analysis, predicting that the “C/C” homozygous genotype of rs6871536 poses a risk for childhood-onset asthma (Table 4).

Both SNP variants, rs2244012 and rs6871536, demonstrated significant associations with childhood-onset asthma susceptibility in the allelic and genotypic models in our studied population. Notably, a similar association of these variants with asthma has been reported in other populations, including German, Danish, and American groups [14, 20, 22].

Analysis using HaploReg revealed that the “G” allele of the rs2244012 variant alters two regulatory motifs: Nrf-2 and Zbtb12. The altered “G” allele slightly increases the binding affinity of the Nrf-2 transcription factor (12.5) relative to the “A” (11.2) reference allele. In contrast, the alternative allele significantly decreases the binding affinity of the transcription factor in the case of Zbtb12 (Table 6). The Nrf-2 transcription factor plays a pivotal role in the regulation of inflammatory genes. It aids in the anti-inflammatory process by mobilizing inflammatory cells. Moreover, Nrf-2 modulates gene expression via the anti-oxidant response element (ARE). Notably, the Nrf2/ARE signaling pathway is critically involved in suppressing inflammatory progression and orchestrating the regulation of anti-inflammatory genes [23]. The Zbtb12 is a recognized transcription factor, and methylation of Zbtb12 Factor 2 (including CpG units 8, 9–10, 16, 21) is positively associated with TNF-⊠ stimulated procoagulant activity, a measure of procoagulant and inflammatory potential of blood cells [24]. The presence of the “G” allele in the rs2244012 variant may lead to alterations in regulatory motifs, potentially disrupting the regulation of the RAD50 gene.

Furthermore, HaploReg analysis indicated that the “C” allele (alternative) of the rs6871536 variant affects the OSF-2 motif, the alternative “C” allele significantly enhances the binding affinity (12.5) of the OSF-2 transcription factor compared to the reference “T” allele binding affinity (0.5) for OSF-2 (Table 6). OSF-2, also known as Periostin, primarily plays a role in osteoblasts and is responsible for bone formation. While Periostin's expression is minimal in most adult tissues, it is markedly elevated at sites of inflammation, tumors, and injury [25]. Notably, increased levels of serum Periostin are consistently observed in conditions like asthma and other allergic diseases [26]. The alteration of the OSF-2/Periostin motif by the “C” allele of the rs6871536 variant might influence the regulation of the *RAD50* gene.

LD analysis for the rs2244012 SNP variant identified 10 single nucleotide variants within the Punjabi population of Lahore, Pakistan (Supplementary Table 1). These high LD SNPs could potentially affect *RAD50* functionality synergistically.

On the other hand, the rs6871536 SNP is in high LD with 21 single nucleotide variants within the same population (Supplementary Table 2). Functional analyses of these high LD variants indicate that they can modify regulatory motifs associated with the regulation of pro/anti-inflammatory genes. These genes either directly or indirectly contribute to asthma's pathogenesis, as elaborated in Supplementary Tables 1 and 2.

Considering the roles of the target SNPs (rs2244012 and rs6871536) of *RAD50* and their associated high LD/proxy SNPs in regulating the *RAD50* gene through changes in regulatory motifs, iťs plausible to infer that even as intronic variants, rs2244012 and rs6871536 may influence *RAD50* gene expression. This could be either independently or in synergy with neighboring single nucleotide variants co-inheriting with these primary SNPs. Understanding the role of these variants as eQTL would be interesting to know further for confirmation if they are really involved in the expression of *RAD50* gene.

Nonetheless, the limited sample size may influence the statistical robustness of the results. Hence, investigations involving larger cohorts and diverse ethnic populations are recommended to further assess the role of these variants in asthma.

## Conclusion

As indicated by GWASs these variants (rs2244012, rs6871536) are significantly linked to asthma susceptibility, our study also identifies a strong association within our study population. Although rs2244012 and rs6871536 are intronic SNP variants, they possess the potential to regulate inflammatory/immunogenic pathways. This regulation can occur independently or in synergy with nearby DNA sequence variants.

## Supplementary Material

This is a list of supplementary files associated with this preprint. Click to download.

• SupplementaryTable1.docx

• SupplementaryTable2.docx

## Figures and Tables

**Figure 1:**
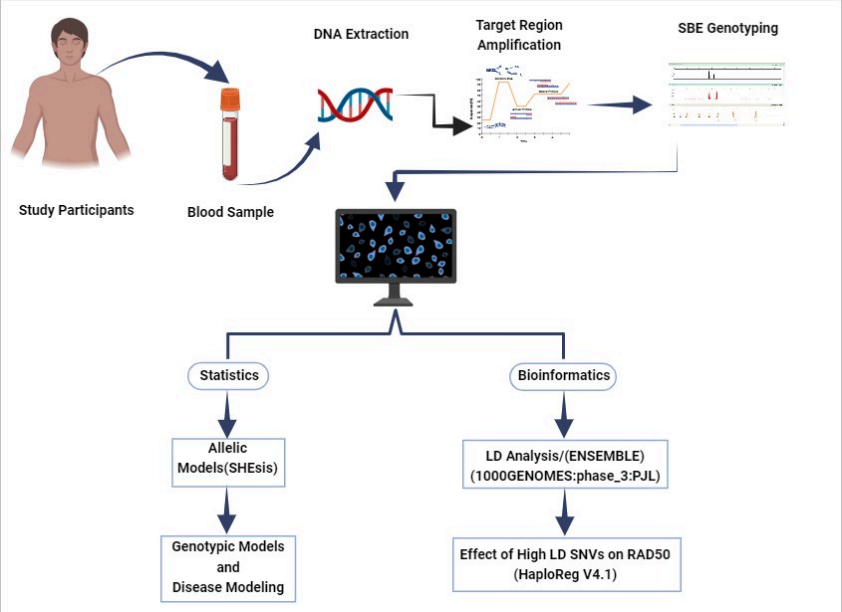
Legend not included with this version.

**Table 1:** Descriptive characteristics of case-control study participants.

Variables	Asthmatics	Healthy Controls
Study Participants	176	179
Mean Age (Years ± SD)	8.91 ± 3.05	11.10 ± 8.86
Sex (Male %)	108 (67.92%)	88 (52.1%)
Residential Area (Urban %)	79 (50.0%)	108 (69.23%)
Parents Inter-relation (Cousin-Marriage)	50 (63.29%)	65 (45.13%)
Mother’s Smoke Exposure during gestation (Yes %)	77 (52.38%)	55 (38.19%)

**Table 2:** Association of SNPs in Allelic Model

SNP	Effect allele	MAF	OR	P-value	FDR_BH
rs2244012	G	0.296	1.80838(1.273 ~ 2.57)	9.22e-04	0.001
rs6871536	C	0.293	1.57504(1.11 ~ 2.25)	0.012	0.012

MAF: Minor allele frequency

**Table 3:** Association of rs2244012 in Multiple Inheritance Models

rs2244012 association with response group (adjusted by sex)						
Model	Genotype	Control	Case	OR [95% CI]	P-value	AIC
Codominant	A/A	66.5%	51.6%	1.00	0.0085	438.6
	A/G	28.1%	38.2%	1.78 (1.09–2.89)		
	G/G	5.4%	10.2%	2.96 (1.22–7.21)		
Dominant	A/A	66.5%	51.6%	1.00	0.0039	437.8
	A/G-G/G	33.5%	48.4%	1.95 (1.23–3.08)		
Recessive	A/A-A/G	94.6%	89.8%	1.00	0.044	442
	G/G	5.4%	10.2%	2.40 (1.00–5.72)		
Overdominant	A/A-G/G	71.9%	61.8%	1.00	0.06	442.6
	A/G	28.1%	38.2%	1.57 (0.98–2.52)		

P-value < 0.05 was considered cut off value for level of significance and lowest Akaike information criterion (**AIC**) test value represents best fit model according to the guidelines of the tool used. Odds ratio is referenced to the reference allele

**Table 4:** Association of rs6871536 in Multiple Inheritance Models

rs6871536 association with response group (adjusted by sex)						
Model	Genotype	Control	Case	OR (95% CI)	P-value	AIC
Codominant	T/T	61.7%	52.9%	1.00	0.06	408.8
	C/T	32.9%	36.6%	1.34 (0.81–2.20)		
	C/C	5.4%	10.5%	2.85 (1.12–7.22)		
Dominant	T/T	61.7%	52.9%	1.00	0.076	409.3
	C/T-C/C	38.3%	47.1%	1.53 (0.95–2.45)		
Recessive	T/T-C/T	94.6%	89.5%	1.00	0.038	408.1
	C/C	5.4%	10.5%	2.55 (1.03–6.32)		
Overdominant	T/T-C/C	67.1%	63.4%	1.00	0.5	412
	C/T	32.9%	36.6%	1.18 (0.73–1.92)		

P-value < 0.05 was considered cut off value for level of significance and lowest Akaike information criterion (**AIC**) test value represents best fit model according to the guidelines of tool used.

**Table 5:** Association of SNPs with covariates

SNP ID	Best Fit Inheritance Model	Sex		Onset Age		Residential Area		Consanguineous Marriage		Pre-natal Smoke Exposure	
		p-Value	Trend	p-Value	Trend	p-Value	Trend	p-Value	Trend	p-Value	Trend
rs2244012	dominant	0.84	N/A	0.66	N/A	0.053	R & SU	0.23	N/A	0.97	N/A
rs6871536	recessive	0.82	N/A	0.90	N/A	0.55	N/A	0.59	N/A	0.41	N/A

SU = Sub urban, R = rural

**Table 6:** LD between RAD-50 gene’s SNPs and HaploReg analysis

Sr.no.	Chr. Position	dbSNP ID	Sequence Consequence	Motifs Changed	TF Binding Affinity Score	LD in “RJL”	Function of Transcription Factor (TF)
1.	5:132565533	rs2244012	intron variant	Nrf-2, Zbtb12	A = 11.2, G = 12.5 A = 9.9 G = 3.1	0.974420	**Nrf-2** [anti-inflammatory response]^1^, **Zbtb12** [DNA methylation, Inflamation] ^2^
2.	5:132634182	rs6871536	intron variant	Osf2	T = 0.5 C = 12.5	0.974420	**Osf2** [Asthma and allergy] ^3^

LD in “PJL”: Linkage Disequilibrium among main SNPs (rs2244012, rs6871536) in “Punjabi in Lahore-Pakistan(1000GENOMES:phase_3:**PJL**)” population.

1 Ahmed, S. M., Luo, L., Namani, A., Wang, X. J. & Tang, X. Nrf2 signaling pathway: Pivotal roles in inflammation. *Biochim Biophys Acta Mol Basis Dis* **1863**, 585–597, doi:10.1016/j.bbadis.2016.11.005 (2017).

2 Noro, F. *et al*. ZBTB12 DNA methylation is associated with coagulation-and inflammation-related blood cellparameters: findings from the Moli-family cohort. *Clinical epigenetics* **11**, 74 (2019).

3 Zielinska-Blizniewska, H. *et al*. Association of the - 33C/G OSF-2 and the 140A/G LF gene polymorphisms with therisk of chronic rhinosinusitis with nasal polyps in a Polish population. *Mol Biol Rep* **39**, 5449–5457, doi:10.1007/s11033-011-1345-6 (2012).

## Data Availability

All data generated or analysed during this study are included in this published article [and its supplementary information files]
